# Supplementary material for: Adenovirus Terminal Protein Contains a Bipartite Nuclear Localisation Signal Essential for Its Import into the Nucleus
Source: Int J Mol Sci. 2021 Mar 24;22(7):3310. doi: 10.3390/ijms22073310 (PMC8036708; doi:10.3390/ijms22073310)
Supplement: Supplementary file 1 [file ijms-22-03310-s001.pdf]

Supporting Information

Adenovirus terminal protein contains a bipartite nuclear localisation signal essential for its import into the nucleus

Hareth A. Al-Wassiti[*1], David R. Thomas[2], Kylie M. Wagstaff[2], Stewart A. Fabb[3], David A. Jans[2], Angus P. Johnston[1], and Colin W. Pouton[*1]

[1] Drug Delivery, Disposition and Dynamics, Monash Institute of Pharmaceutical Sciences, Monash University, Melbourne, Australia
[2] Department of Biochemistry and Molecular Biology, Monash Biomedicine Discovery Institute, Melbourne, Australia
[3] Drug Discovery Biology, Monash Institute of Pharmaceutical Sciences, Monash University, Melbourne, Australia
* Correspondence: Colin.Pouton@monash.edu; Harry.al-wassiti@monash.edu

List of Fugures and Tables:

1. Figure S1. **Expression and localisation of pTP, TP, pMax, F1-F4 fragments in 293 Cells**.
2. Figure S2. **Refolding screening assay of TP using different buffers and additives**.
3. Figure S3. **Conjugation of AF594 to TP and the associated tag**.
4. Figure S4**. Used as control, BSA-NLS nuclear accumulation is inhibited by Ivermectin treatment.**
5. Table S1. Oligonucleotides used to generate fragments.
6. Table S2. Oligonucleotides used for sequencing and GFP backbone amplification.
7. Table S3. Fragments N/C means, SD, SEM and number of cells.
8. Table S4. HeLa Post Hoc Tukey's comparisons.
9. Table S5. 293A Post Hoc Tukey's comparisons.
10. Table S6. Hela and 293 post hoc comparison of mutations and deletion fragments.
11. Table S7. Hela post hoc comparison between drug treatments using microinjection.
12. Table S8 T-test (Welch test) between pTP and GFP.

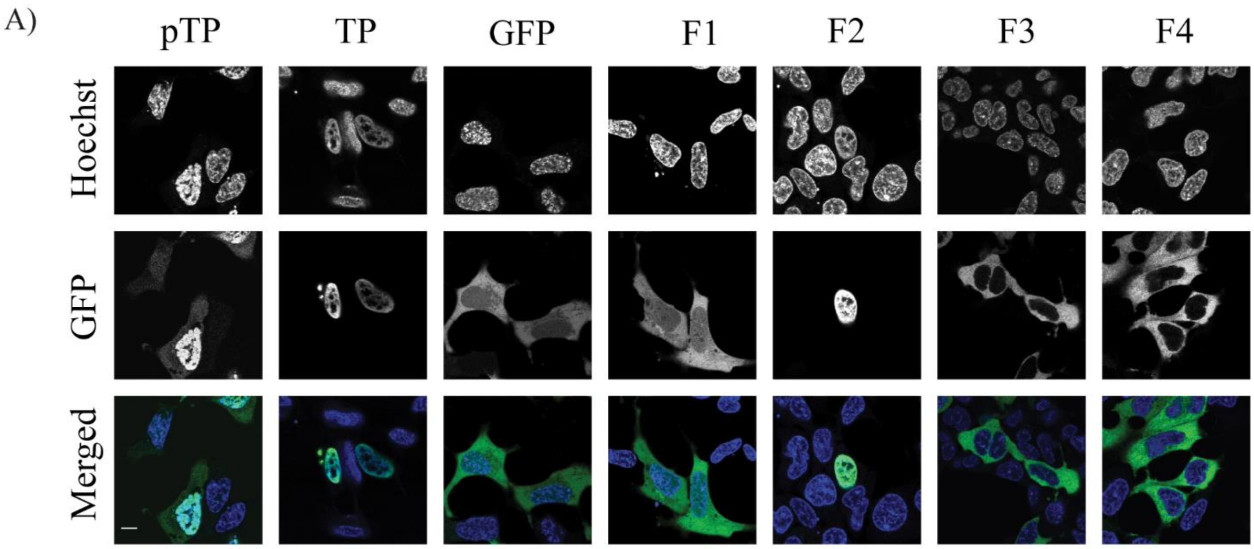

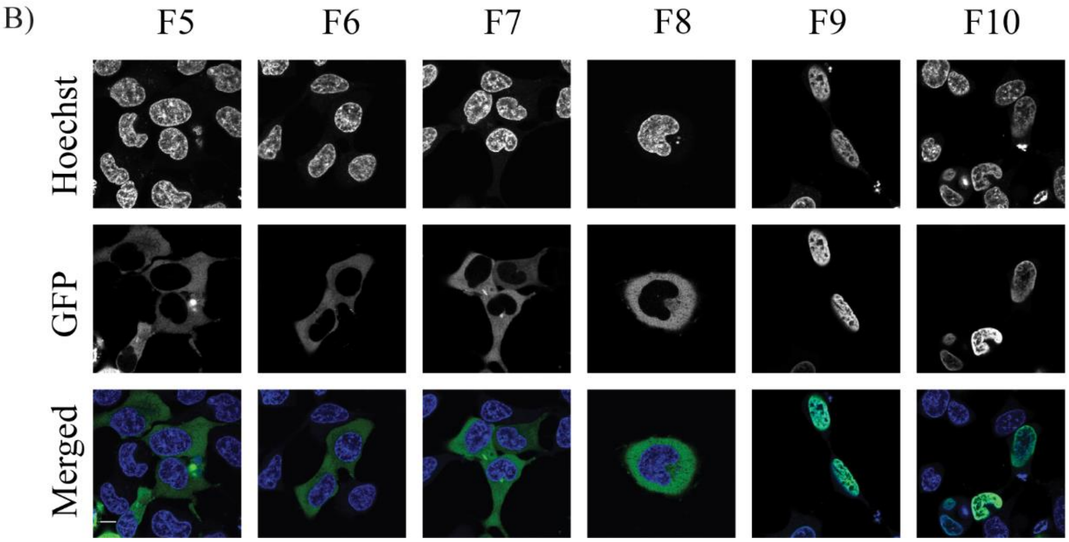

Figure S1. Expression and localisation of pTP, TP, pMax, F1-F4 fragments in 293 Cells. Plasmids containing the fragments were transfected into 293 cells. (A)Fragments: 1-4, TP and pTP. (B) F5-F10. Representative images of GFP localisation for each fragment are shown with GFP, Hoechst nuclear stain and merged at the bottom row as described in figure 2. Bar = 10µm.

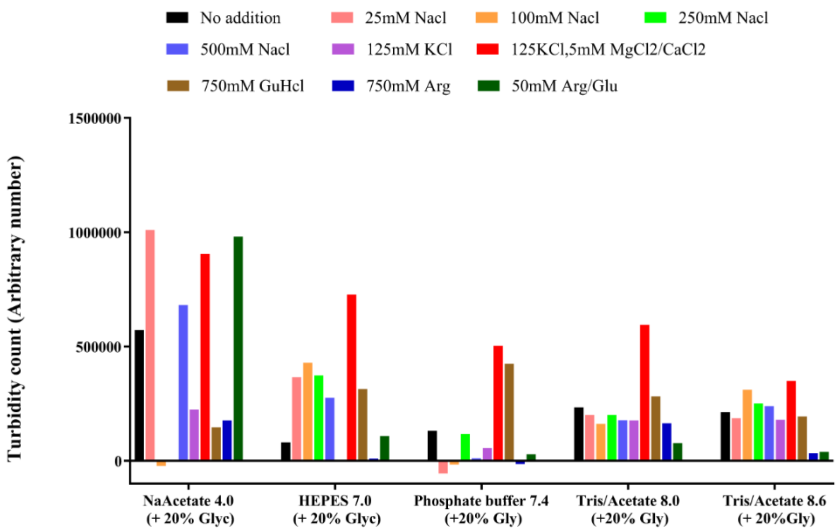

Figure S2. Refolding screening assay of TP using different buffers and additives.TP (in 7M urea) was dialysed at 4°C in different buffers with/without additives (salts and stabilisers). Next day, turbidity using nephelometer was measured at the same time and plotted as an arbitrary number. All conditions had 20% Glycerol.

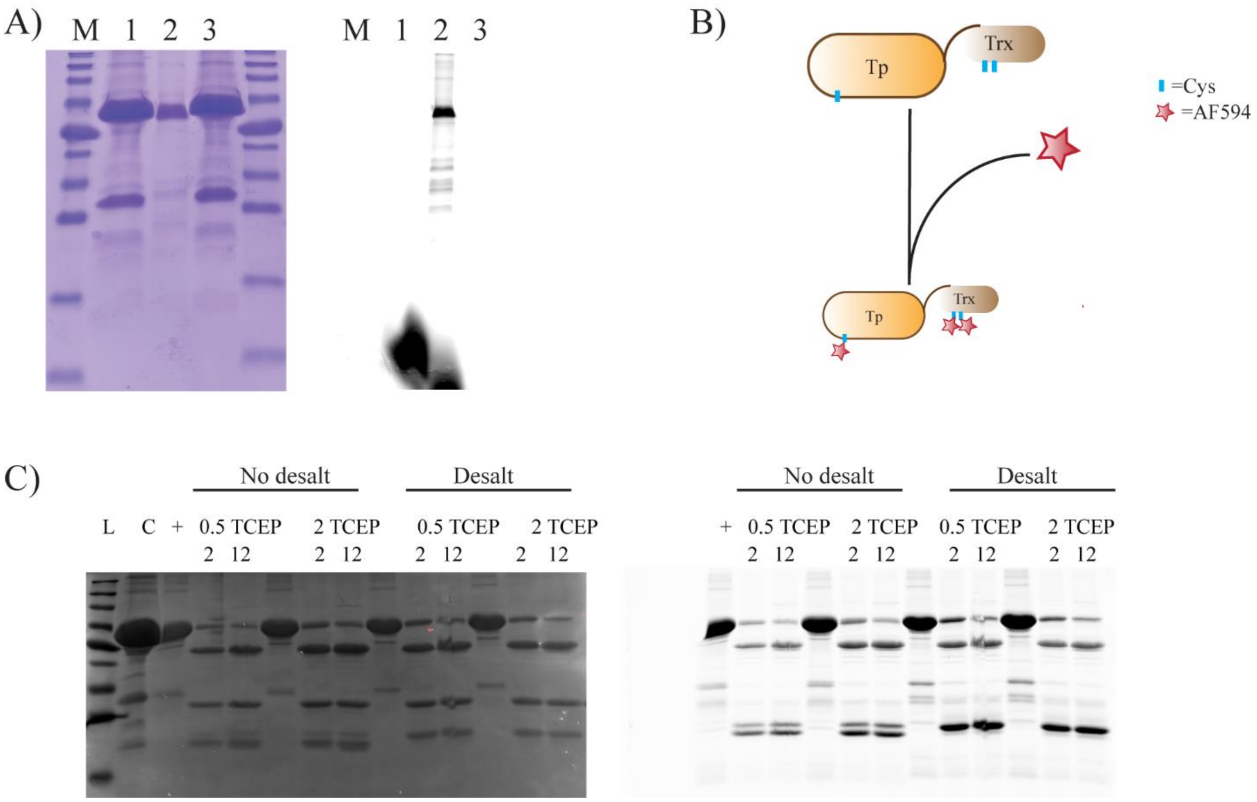

Figure S3. Conjugation of AF594 to TP and the associated tag. (A) SDS-page (left) and fluorescence light specific to AF594 using Typhoon imager (GE healthcare) showed on the right. 1: TP and AF-594-maleimide mixed and immediately loaded on SDS-page; 2: TP was conjugated with AF-594-maleimide as described in the methods; 3: unconjugated TP. **B)** schematic representation of the proposed conjugation locations corresponding to the three Cysteines found on the protein. **C)** Cleaving test showing the level of conjugation with AF594 at the three sites was used to optimise the conditions of conjugation. L: ladder; C: control no conjugation; +: TP control conjugated with AF594 (pre-incubated with 2mM TCEP); and in the rest of conditions TP-Trx was pre-incubated with either 0.5 or 2mM TCEP for 30 minutes, and TCEP desalted or not removed followed by incubation with AF594. After labelling and label removal from the conditions described above, TP-Trx was TEV-digested for 2 or 12 hours as indicated in the bottom row. Left: SDS page image. Right: Fluorescent gel image.

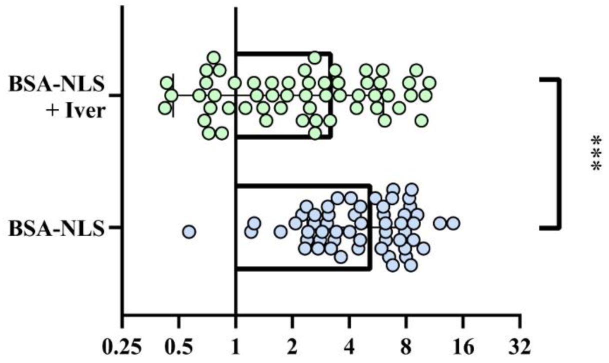

Figure S4. Used as control, BSA-NLS nuclear accumulation is inhibited by Ivermectin treatment. Ivermectin (30µM) was incubated with cells for 3 hours, followed by microinjection. N_f/C_f was calculated as described in the Methods section and data presented mean±SD. Data were derived from at least two independent biological replicates ($n_{cells} \geq 56$). Welch's t-test shows a significant difference between drug-treated and untreated p-value of 0.0003.

Table S1. Oligonucleotides used to generate fragments (5’-3’)

Oligo #	Directi on	Shared by	Sequence 5’-3’
**1. Ol 1**	FWR	pTP, F1,F2, F3, F4	CCTTCGCCAGATCTCGAGCTGAGCACTTTTTGCCGCTG CGC
**2. Ol 2**	RVS	pTP,TP,F5,F6	TTGTCCAAACTCATCGAGCTCTAAAAGCGGTGACGCG GGCG
**3. Ol 3**	FWR	TP, F9, F10	CCTTCGCCAGATCTCGAGCTGTCTTCCAACTGCGCCCC CG
**4. Ol 4**	RVS	F1	TTGTCCAAACTCATCGAGCTACTAAGTGTCGCGCACCT CGCG
**5. Ol 5**	RVS	F2	TTGTCCAAACTCATCGAGCTACTAGACACGGCGGCGA CGACG
**6. Ol 6**	RVS	F3	TTGTCCAAACTCATCGAGCTACTAGATCATCTCCCCGC GGCGAC
**7. Ol 7**	RVS	F4	TTGTCCAAACTCATCGAGCTACTAGGTGACGGCGCGG CCGTTC
**8. Ol 8**	FWR	F5, F8	CCTTCGCCAGATCTCGAGCTCGCTTTGTCGACCGCCTC CC
**9. Ol 9**	FWR	F7	CCTTCGCCAGATCTCGAGCTCCAGAAGAAGAAGAAGG GGAGGCC
**10. Ol 10**	FWR	F6	CCTTCGCCAGATCTCGAGCTTCAGGAGACGTGCAGGA GATTTTGC
**11. Ol 11**	RVS	F7, F8, F9	TTGTCCAAACTCATCGAGCTACTAGAGAGGGGGCAGG GGCACGTC
**12. Ol 12**	RVS	F10	TTGTCCAAACTCATCGAGCTACTAGTTGTCTTGATAGG CGATCTCGGCCATG
**13. Mut1F**	FWD	MUT1	TCTCGTGGTGAGATGATCGAGCGCTTTGTCG
**14. Mut1R**	RVS	MUT1	AGCAGACATGGTCTCGGTGACGG
**15. Mut2F**	FWD	MUT2	TCTGTCCCACCTCCTCCACCGCCGCCAGAAGAAG
**16. Mut2R**	RVS	MUT2	GCGAGTAGATGTAACCGGGAGGCGGTCGACAAAGC
**17. Mut3F**	FWD	MUT3	CACACTTTTTAGAGCTCGATGAGTTTG
**18. Mut3R**	RVS	MUT3	AGTCGGTGTAGCACCCGGAGGTAG
**19. Del1F**	FWD	DEL1	CCGCCGCCAGAAGAAGAAGAAGG
**20. Del1R**	RVS	DEL1	GACAAAGCGCTCGATCATCTCC

Table S2. Oligonucleotides used for sequencing and GFP backbone amplification (5’-3’)

Given name	Sequence
**pMaxF**	CTCGATGAGTTTGGACAAACCAC
**pMaxR**	CTCGAGATCTGGCGAAGGC
**Frag-SeqFV2**	CCA GAT TCA GGA GAT CAA CCG C
**Frag-SeqF**	ACA GCC ACA TGC ACT TCA AGA GC

Table S3. Fragments N_f/C_f means, SD, SEM and number of cells.

**Fragment/Mutant**	**Mean**	**SD**	**SEM**	**N**
GFP 293	0.79	0.31	0.07	19
GFP Hela	0.83	0.17	0.04	22
pTP 293	3.19	3.34	0.70	23
pTP Hela	4.91	4.58	0.77	35
TP 293	23.27	15.90	3.75	18
TP Hela	27.12	25.18	4.60	30
F1 293	0.73	0.22	0.04	28
F1 Hela	1.72	0.78	0.17	20
F2 293	8.58	9.99	2.08	23
F2 Hela	14.18	17.10	2.85	36
F3 293	0.32	0.10	0.02	26
F3 Hela	0.57	0.18	0.04	17
F4 293	0.32	0.13	0.03	21
F4 Hela	0.48	0.14	0.03	26
F5 293	0.24	0.09	0.02	28
F5 Hela	0.78	0.48	0.10	23
F6 293	0.34	0.18	0.04	21
F6 Hela	0.65	0.56	0.10	32
F7 293	0.45	0.24	0.05	20
F7 Hela	0.90	0.26	0.07	15
F8 293	0.27	0.20	0.04	24
F8 Hela	0.44	0.16	0.04	17
F9 293	13.67	12.20	2.27	29
F9 Hela	21.66	21.05	3.78	31
F10 293	19.74	18.31	4.58	16
F10 Hela	17.94	19.84	3.31	36
Mut-1 293	0.37	0.13	0.02	35
Mut-1 Hela	0.48	0.18	0.03	34
Mut-2 293	1.90	0.99	0.23	18
Mut-2 Hela	1.68	0.67	0.15	21
Mut-3 293	14.79	15.34	3.01	26
Mut-3 Hela	17.78	17.38	3.34	27
Del 293	0.36	0.15	0.03	27
Del Hela	0.43	0.13	0.03	25

SD: standard deviation; SEM: standard error of the mean, N: number of cells.

Table S4 HeLa Post-Hoc Tukey's comparisons. * = p-value<0.05; ** = p-value <0.01; *** = p-value<0.001; **** = p-value<0.0001.

Tukey's multiple comparisons test	Significant?	Summary	P Value
F1 Hela vs. F2 Hela	Yes	*	0.0413
F1 Hela vs. F3 Hela	No	ns	>0.9999
F1 Hela vs. F4 Hela	No	ns	>0.9999
F1 Hela vs. F5 Hela	No	ns	>0.9999
F1 Hela vs. F6 Hela	No	ns	>0.9999
F1 Hela vs. F7 Hela	No	ns	>0.9999
F1 Hela vs. F8 Hela	No	ns	>0.9999
F1 Hela vs. F9 Hela	Yes	****	<0.0001
F1 Hela vs. F10 Hela	Yes	***	0.0009
F1 Hela vs. GFP Hela	No	ns	>0.9999
F1 Hela vs. pTP Hela	No	ns	0.9997
F1 Hela vs. TP Hela	Yes	****	<0.0001
F2 Hela vs. F3 Hela	Yes	*	0.0281
F2 Hela vs. F4 Hela	Yes	**	0.0042
F2 Hela vs. F5 Hela	Yes	*	0.01
F2 Hela vs. F6 Hela	Yes	**	0.002
F2 Hela vs. F7 Hela	No	ns	0.0579
F2 Hela vs. F8 Hela	Yes	*	0.0252
F2 Hela vs. F9 Hela	No	ns	0.5006
F2 Hela vs. F10 Hela	No	ns	0.9919
F2 Hela vs. GFP Hela	Yes	*	0.0126
F2 Hela vs. pTP Hela	No	ns	0.138
F2 Hela vs. TP Hela	Yes	**	0.0055
F3 Hela vs. F4 Hela	No	ns	>0.9999
F3 Hela vs. F5 Hela	No	ns	>0.9999
F3 Hela vs. F6 Hela	No	ns	>0.9999
F3 Hela vs. F7 Hela	No	ns	>0.9999
F3 Hela vs. F8 Hela	No	ns	>0.9999
F3 Hela vs. F9 Hela	Yes	****	<0.0001
F3 Hela vs. F10 Hela	Yes	***	0.0007
F3 Hela vs. GFP Hela	No	ns	>0.9999
F3 Hela vs. pTP Hela	No	ns	0.9962
F3 Hela vs. TP Hela	Yes	****	<0.0001
F4 Hela vs. F5 Hela	No	ns	>0.9999
F4 Hela vs. F6 Hela	No	ns	>0.9999
F4 Hela vs. F7 Hela	No	ns	>0.9999
F4 Hela vs. F8 Hela	No	ns	>0.9999
F4 Hela vs. F9 Hela	Yes	****	<0.0001
F4 Hela vs. F10 Hela	Yes	****	<0.0001
F4 Hela vs. GFP Hela	No	ns	>0.9999

F4 Hela vs. pTP Hela	No	ns	0.9853
F4 Hela vs. TP Hela	Yes	****	<0.0001
F5 Hela vs. F6 Hela	No	ns	>0.9999
F5 Hela vs. F7 Hela	No	ns	>0.9999
F5 Hela vs. F8 Hela	No	ns	>0.9999
F5 Hela vs. F9 Hela	Yes	****	<0.0001
F5 Hela vs. F10 Hela	Yes	***	0.0001
F5 Hela vs. GFP Hela	No	ns	>0.9999
F5 Hela vs. pTP Hela	No	ns	0.9942
F5 Hela vs. TP Hela	Yes	****	<0.0001
F6 Hela vs. F7 Hela	No	ns	>0.9999
F6 Hela vs. F8 Hela	No	ns	>0.9999
F6 Hela vs. F9 Hela	Yes	****	<0.0001
F6 Hela vs. F10 Hela	Yes	****	<0.0001
F6 Hela vs. GFP Hela	No	ns	>0.9999
F6 Hela vs. pTP Hela	No	ns	0.9831
F6 Hela vs. TP Hela	Yes	****	<0.0001
F7 Hela vs. F8 Hela	No	ns	>0.9999
F7 Hela vs. F9 Hela	Yes	****	<0.0001
F7 Hela vs. F10 Hela	Yes	**	0.0022
F7 Hela vs. GFP Hela	No	ns	>0.9999
F7 Hela vs. pTP Hela	No	ns	0.9988
F7 Hela vs. TP Hela	Yes	****	<0.0001
F8 Hela vs. F9 Hela	Yes	****	<0.0001
F8 Hela vs. F10 Hela	Yes	***	0.0006
F8 Hela vs. GFP Hela	No	ns	>0.9999
F8 Hela vs. pTP Hela	No	ns	0.9951
F8 Hela vs. TP Hela	Yes	****	<0.0001
F9 Hela vs. F10 Hela	No	ns	0.995
F9 Hela vs. GFP Hela	Yes	****	<0.0001
F9 Hela vs. pTP Hela	Yes	****	<0.0001
F9 Hela vs. TP Hela	No	ns	0.9219
F10 Hela vs. GFP Hela	Yes	***	0.0002
F10 Hela vs. pTP Hela	Yes	**	0.0026
F10 Hela vs. TP Hela	No	ns	0.1959
GFP Hela vs. pTP Hela	No	ns	0.9954
GFP Hela vs. TP Hela	Yes	****	<0.0001
pTP Hela vs. TP Hela	Yes	****	<0.0001

Table S5. 293A PostHoc Tukey's comparisons.
* = p-value<0.05; ** = p-value <0.01; *** = p-value<0.001; **** = p-value<0.0001.

Tukey's multiple comparisons test	Significant?	Summary	p-Value
F1 293 vs. F2 293	Yes	*	0.0148
F1 293 vs. F3 293	No	ns	>0.9999
F1 293 vs. F4 293	No	ns	>0.9999
F1 293 vs. F5 293	No	ns	>0.9999
F1 293 vs. F6 293	No	ns	>0.9999
F1 293 vs. F7 293	No	ns	>0.9999
F1 293 vs. F8 293	No	ns	>0.9999
F1 293 vs. F9 293	Yes	****	<0.0001
F1 293 vs. F10 293	Yes	****	<0.0001
F1 293 vs. GFP 293	No	ns	>0.9999
F1 293 vs. pTP 293	No	ns	0.9945
F1 293 vs. TP 293	Yes	****	<0.0001
F2 293 vs. F3 293	Yes	**	0.0094
F2 293 vs. F4 293	Yes	*	0.019
F2 293 vs. F5 293	Yes	**	0.0064
F2 293 vs. F6 293	Yes	*	0.0195
F2 293 vs. F7 293	Yes	*	0.0268
F2 293 vs. F8 293	Yes	*	0.0113
F2 293 vs. F9 293	No	ns	0.4249
F2 293 vs. F10 293	Yes	***	0.0005
F2 293 vs. GFP 293	Yes	*	0.0492
F2 293 vs. pTP 293	No	ns	0.4223
F2 293 vs. TP 293	Yes	****	<0.0001
F3 293 vs. F4 293	No	ns	>0.9999
F3 293 vs. F5 293	No	ns	>0.9999
F3 293 vs. F6 293	No	ns	>0.9999
F3 293 vs. F7 293	No	ns	>0.9999
F3 293 vs. F8 293	No	ns	>0.9999
F3 293 vs. F9 293	Yes	****	<0.0001
F3 293 vs. F10 293	Yes	****	<0.0001
F3 293 vs. GFP 293	No	ns	>0.9999
F3 293 vs. pTP 293	No	ns	0.982
F3 293 vs. TP 293	Yes	****	<0.0001
F4 293 vs. F5 293	No	ns	>0.9999
F4 293 vs. F6 293	No	ns	>0.9999
F4 293 vs. F7 293	No	ns	>0.9999
F4 293 vs. F8 293	No	ns	>0.9999
F4 293 vs. F9 293	Yes	****	<0.0001
F4 293 vs. F10 293	Yes	****	<0.0001
F4 293 vs. GFP 293	No	ns	>0.9999
F4 293 vs. pTP 293	No	ns	0.9886
F4 293 vs. TP 293	Yes	****	<0.0001

F5 293 vs. F6 293	No	ns	>0.9999
F5 293 vs. F7 293	No	ns	>0.9999
F5 293 vs. F8 293	No	ns	>0.9999
F5 293 vs. F9 293	Yes	****	<0.0001
F5 293 vs. F10 293	Yes	****	<0.0001
F5 293 vs. GFP 293	No	ns	>0.9999
F5 293 vs. pTP 293	No	ns	0.9742
F5 293 vs. TP 293	Yes	****	<0.0001
F6 293 vs. F7 293	No	ns	>0.9999
F6 293 vs. F8 293	No	ns	>0.9999
F6 293 vs. F9 293	Yes	****	<0.0001
F6 293 vs. F10 293	Yes	****	<0.0001
F6 293 vs. GFP 293	No	ns	>0.9999
F6 293 vs. pTP 293	No	ns	0.9892
F6 293 vs. TP 293	Yes	****	<0.0001
F7 293 vs. F8 293	No	ns	>0.9999
F7 293 vs. F9 293	Yes	****	<0.0001
F7 293 vs. F10 293	Yes	****	<0.0001
F7 293 vs. GFP 293	No	ns	>0.9999
F7 293 vs. pTP 293	No	ns	0.9932
F7 293 vs. TP 293	Yes	****	<0.0001
F8 293 vs. F9 293	Yes	****	<0.0001
F8 293 vs. F10 293	Yes	****	<0.0001
F8 293 vs. GFP 293	No	ns	>0.9999
F8 293 vs. pTP 293	No	ns	0.9822
F8 293 vs. TP 293	Yes	****	<0.0001
F9 293 vs. F10 293	No	ns	0.3181
F9 293 vs. GFP 293	Yes	****	<0.0001
F9 293 vs. pTP 293	Yes	****	<0.0001
F9 293 vs. TP 293	Yes	**	0.0019
F10 293 vs. GFP 293	Yes	****	<0.0001
F10 293 vs. pTP 293	Yes	****	<0.0001
F10 293 vs. TP 293	No	ns	0.9778
GFP 293 vs. pTP 293	No	ns	0.9983
GFP 293 vs. TP 293	Yes	****	<0.0001
pTP 293 vs. TP 293	Yes	****	<0.0001

Table S6. Hela and 293 post hoc comparison with mutations and deletion fragments. * = p-value<0.05; ** = p-value <0.01; *** = p-value<0.001; **** = p-value<0.0001.

Tukey's multiple comparisons test	Significant?	Summary	p-Value
GFPHela vs. pTP Hela	No	ns	0.8729
GFPHela vs. TP Hela	Yes	****	<0.0001

GFPHela vs. Mut-1 Hela	No	ns	>0.9999
GFPHela vs. Mut-2 Hela	No	ns	>0.9999
GFPHela vs. Mut-3 Hela	Yes	****	<0.0001
GFPHela vs. Del Hela	No	ns	>0.9999
pTP Hela vs. TP Hela	Yes	****	<0.0001
pTP Hela vs. Mut-1 Hela	No	ns	0.7246
pTP Hela vs. Mut-2 Hela	No	ns	0.9586
pTP Hela vs. Mut-3 Hela	Yes	***	0.0009
pTP Hela vs. Del Hela	No	ns	0.787
TP Hela vs. Mut-1 Hela	Yes	****	<0.0001
TP Hela vs. Mut-2 Hela	Yes	****	<0.0001
TP Hela vs. Mut-3 Hela	No	ns	0.0573
TP Hela vs. Del Hela	Yes	****	<0.0001
Mut-1 Hela vs. Mut-2 Hela	No	ns	0.9998
Mut-1 Hela vs. Mut-3 Hela	Yes	****	<0.0001
Mut-1 Hela vs. Del Hela	No	ns	>0.9999
Mut-2 Hela vs. Mut-3 Hela	Yes	***	0.0001
Mut-2 Hela vs. Del Hela	No	ns	0.9998
Mut-3 Hela vs. Del Hela	Yes	****	<0.0001
GFP 293 vs. pTP 293	No	ns	0.9628
GFP 293 vs. TP 293	Yes	****	<0.0001
GFP 293 vs. Mut-1 293	No	ns	>0.9999
GFP 293 vs. Mut-2 293	No	ns	0.9996
GFP 293 vs. Mut-3 293	Yes	****	<0.0001
GFP 293 vs. Del 293	No	ns	>0.9999
pTP 293 vs. TP 293	Yes	****	<0.0001
pTP 293 vs. Mut-1 293	No	ns	0.8526
pTP 293 vs. Mut-2 293	No	ns	0.9988
pTP 293 vs. Mut-3 293	Yes	****	<0.0001
pTP 293 vs. Del 293	No	ns	0.8814
TP 293 vs. Mut-1 293	Yes	****	<0.0001
TP 293 vs. Mut-2 293	Yes	****	<0.0001
TP 293 vs. Mut-3 293	Yes	*	0.014
TP 293 vs. Del 293	Yes	****	<0.0001
Mut-1 293 vs. Mut-2 293	No	ns	0.9948
Mut-1 293 vs. Mut-3 293	Yes	****	<0.0001
Mut-1 293 vs. Del 293	No	ns	>0.9999
Mut-2 293 vs. Mut-3 293	Yes	****	<0.0001
Mut-2 293 vs. Del 293	No	ns	0.9959
Mut-3 293 vs. Del 293	Yes	****	<0.0001

Table S7

post hoc comparison of microinjection and drug treatments. Analysis corresponds to Fig. 7B. * = p-value<0.05; ** = p-value <0.01; *** = p-value<0.001; **** = p-value<0.0001.

Tukey's multiple comparisons tests	Significant?	Summary	p-Value
TP vs. TP+Iver	Yes	****	<0.0001
TP vs. TP+Impz	Yes	***	0.0002
TP vs. TP+LepB	No	ns	0.2024
TP+Iver vs. TP+Impz	No	ns	0.5567
TP+Iver vs. TP+LepB	Yes	***	0.0005
TP+Impz vs. TP+LepB	No	ns	0.0753

Table S8

The t-test (Welch test) between pTP and GFP transfection of immortal cell lines. The analysis corresponds to Fig 2.

**Welch's t-test**	**Means**	**Significant**	**pValue**
**pTp vs. GFP (293A)**	**pTP** = 0.7917 **vs GFP=** 3.189	**YES**	0.0024
**pTp vs. GFP (Hela)**	**pTP** = 0.8254 **vs GFP=** 4.912	**YES**	<0.0001
